# Patient & staff perceptions of animal-assisted therapy in psychiatric rehabilitation

**DOI:** 10.1192/bjo.2021.578

**Published:** 2021-06-18

**Authors:** Laura Sallette, David King, Sian Cowton-Williams, Rajesh Mohan

**Affiliations:** 1 Heather Close Rehabilitation Unit, South London and Maudsley NHS Foundation trust; 2 King's college london

## Abstract

**Aims:**

To study patients' subjective experiences of having access to a therapy dog and to assess the staff perception of the impact of pet therapy. Hypothesis: pet therapy services are acceptable for rehabilitation patients.

**Background:**

Animal-assisted therapy (AAT) is the supervised use of an animal in a therapeutic setting to help in the treatment of physical or psychological disorders in humans. The use of dogs in the context of AAT - ‘dog therapy’ (DT) - has been piloted in the context of stroke rehabilitation; schizophrenia in elderly patients; depression, loneliness and anxiety in elderly patients; Alzheimer's disease; symptom reduction in PTSD; cognitive impairment; and dementia. The impact of pet therapy in long term psychosis care has not been adequately assessed.

**Method:**

This feasibility pilot study used questionnaires to assess patient (n = 12) and staff (n = 10) perceptions of dog therapy in an in-patient psychiatric rehabilitation setting. 24 patients on a rehabilitation ward with complex psychosis were offered the opportunity to interact with ‘Nugget,’ a corgi trained in the United States as a ‘therapy dog.’ A ‘patient questionnaire’ (PQ) and a ‘staff questionnaire’ (SQ) assessed the acceptability and self-rated benefits of the intervention.

**Result:**

All patients (100%) rated highly on the enjoyment, anxiety, calmness, and comfort domains during the dog therapy, and expressed willingness to receive further sessions in the future. The SQ measured staff perceptions of patients’ engagement, enjoyment, comfort and emotional response to the therapy. 100% of staff rated highly on all questions and thought the interventions had recovery value. Engagement was one key factor noted in the feedback. There were no reported adverse reactions to the intervention.

**Conclusion:**

Our preliminary results showed high acceptability and perceived value for Animal assisted therapy in a psychiatric rehabilitation setting. Given the impact of social isolation and need for connectedness, we recommend access to pet therapy where possible to be integrated into individual recovery programmes.

